# Association between depression scores and comprehensive geriatric assessment and frailty in geriatric outpatients with somatic complaints: an observational cross-sectional study

**DOI:** 10.55730/1300-0144.5365

**Published:** 2022-02-05

**Authors:** Hande SELVİ ÖZTORUN, Bilge GÖZÜKARA, Remzi BAHŞİ, Tuğba TURGUT, Deniz Mut SÜRMELİ, Çağlar COŞARDERELİOĞLU SEÇER, Volkan ATMIŞ, Ahmet YALÇIN, Sevgi ARAS, Murat VARLI

**Affiliations:** 1 Department of Geriatrics, Ankara City Hospital, Ankara, Turkey; 2 Department of Internal Medicine, Faculty of Medicine, Ankara University, Ankara, Turkey; 3 Department of Geriatrics, Faculty of Medicine, Ankara University, Ankara, Turkey; 4 Department of Geriatrics, Antalya Training and Research Hospital, Antalya, Turkey

**Keywords:** Geriatric depression scores, comprehensive geriatric assessment, frailty, functionality, sleep disorders

## Abstract

**Background/aim:**

Depression is the most common psychiatric problem in older individuals. In some countries, the common approach is to ignore psychiatric disorders. This study aimed to reveal the importance of newly diagnosed high depression scores in the geriatric population admitted to outpatient clinics with somatic complaints.

**Materials and methods:**

Patients who did not have a previous diagnosis of a psychiatric disorder and were not receiving treatment were included in the study. A comprehensive geriatric evaluation of 235 elderly patients was performed using established assessment tests. The time and quality of sleep and the Clinical Frailty Scores (CFSs) were documented.

**Results:**

The mean age of the 235 patients was 73.6 ± 6.39 years, 65.5% (n = 154) were women, and 34.9% (n = 81) had a geriatric depression score ≥ 5. In the higher depression rating scores group, the Lawton-Brody, Mini-Mental State Examination (MMSE), and Mini Nutritional Assessment (MNA-SF) scores were low (p = 0.010, p < 0.001, p = 0.003). Sleep duration was short, and sleep quality was poor (p = 0.042, p = 0.006). The CFSs were high. (p = 0.035) According to the regression analysis results, the MMSE, MNA-SF and CFS predicted higher depression scores significantly (p = 0.048, ß = .892; p = 0.045, ß = .661; p = 0.045, ß = 1.245).

**Conclusion:**

Depression scores in older people may be associated with not only mood but also the functionality. As with other geriatric syndromes, symptoms in depression may be atypical rather than typical.

## 1. Introduction

Depression is characterized by depressed mood, loss of interest and pleasure, feelings of guilt or low self-worth, impaired sleep and appetite, low energy, and poor concentration. The World Health Organization (WHO) declared depression to be a major contributor to disability in the world [1]. Geriatric depression, also known as ‘late-life depression’, describes depression manifesting after 60 years of age. Depression is the most common psychiatric problem among older individuals.

According to the latest WHO report, the prevalence rates of depression vary by age, tending to peak in older adulthood (over 7.5% and 5.5% in 55–74-year-old women and men, respectively). Another study conducted in the USA and Australia reported a 9.8% rate [1,2]. However, studies from many developing countries report that this figure can be as high as 30% [3–5].

There are many issues in making the diagnosis of geriatric depression. One of the issues is the incongruity of the tests used for making the diagnosis of depression across different studies in the literature. Another issue is the low level of depression awareness both in society and among physicians, especially in developing countries [6–9]. Furthermore, the presence of multiple chronic diseases causes either failure in identifying depression or the symptoms of depression being attributed to comorbid diseases, leaving depression untreated in this patient population in outpatient clinics of medical specialties other than psychiatry.

In some countries, including Turkey, the predominant approach is to ignore psychiatric disorders and to consider them a low priority for seeing a physician [7]. Many patients with psychiatric disorders consider seeing a psychiatrist to be shameful, or they think they would be blamed by the community, even if it helps them obtain insight into their condition [6]. Consequently, the patient may develop somatisation and consult an internal medicine specialist or a physician of a respective specialty depending on the type of generated somatic complaints. This can lead to a patient overflow in the internal medicine outpatient clinics due to unidentified causes of diseases and numerous diagnostic tests. This situation confuses physicians who have received no training on depressive mood and other psychiatric disorders [8].

Diagnosis of depression in old age is complex, but its consequences are critical. Old-age depression increases mortality and unfavourably affects the well-being and daily functioning of older individuals. It impairs patient compliance to the treatment of chronic diseases. It affects the quality of life negatively. Moreover, it even increases suicidal tendencies [10].

The primary purpose of physicians dealing with older individuals is to ensure that they continue to function throughout their lives. One of the major targets of geriatrics is to identify the factors causing negative outcomes using comprehensive geriatric tests and to provide treatment when possible. Depressive mood negatively affects the activities of daily living by creating cognitive disability in patients. When left untreated, depression creates not only cognitive but functional disability as well.

This study aimed to reveal the importance of high depression scores in the geriatric population admitted to geriatric outpatient clinics with somatic complaints. We wanted to determine the association between depression scores and functionality (basic and instrumental life activities, gait speed, cognition, nutritional status, number of falls, sleep time, and quality and frailty), which has an impact on the quality of life of older people.

## 2. Materials and methods

### 2.1. Patient selection and patient information

This study was conducted in Ankara, Turkey, with patients admitted to the geriatric outpatient clinic. Eligible and voluntary patients admitted to the geriatric outpatient clinic in the period from November 2018 to February 2019 were included in this cross-sectional study. The participants included in the study were patients over 65 years of age who were admitted to the geriatric outpatient clinic with any complaints and in accordance with the study criteria. Patients who were too disabled to complete a comprehensive geriatric assessment, who had dementia with a Mini-Mental State Examination (MMSE) score below 15, or who were known to have a previous diagnosis of depression and were receiving treatment were excluded from the study. Dementia patients with an MMSE score greater than 15 who could communicate and complete the tests were included in the study. The number of patients included in the study after applying the exclusion criteria is shown in Figure. The sociodemographic characteristics and disease information of the patients were recorded (Table 1). The laboratory values of the patients were documented (Table 2).

### 2.2. Comprehensive geriatric assessment

Comprehensive geriatric assessment tests included the Katz Index of Activities of Daily Living (Katz ADL), the Lawton-Brody Instrumental Activities of Daily Living Scale (LB-IADL), the MMSE, the Geriatric Depression Scale (15-item short form) (GDS-15), and the Mini Nutritional Assessment-Short-Form (MNA-SF). Activities of daily living were assessed using the Katz ADL, which assesses dressing, bathing, toileting, transferring, feeding, and continence. It assigns 0 or 1 point for each activity for a total score of 0–6 points [11].

Instrumental activities of daily living were assessed with the LB-IADL. In a range of 0–8 points with either zero or one point for each activity, this scale assesses the following: the ability to use the telephone, shopping, food preparation, housekeeping, laundry, mode of transportation, responsibility for own medications, and ability to handle finances [12]. Cognitive functions were examined with the MMSE. This test grades cognitive functions in a range of 30 points, with lower scores indicating impairment in cognitive functions [13]. The GDS-15 was used to assess the severity of depressive mood [14]. The nutritional status of the patients was evaluated with the MNA-SF [15]. With proven reliability and validity in Turkish, the maximum possible score of the MNA-SF is 14. A score from 0 to 7 points indicates malnutrition, 8 to 11 points indicates a risk of malnutrition, and 12 to 14 points indicates normal nutrition [16]. Muscle strength was assessed as grip strength using an electronic hand dynamometer (GRIP-D digital grip dynamometer; Takei, Tokyo, Japan). Grip strength was measured when the arm was in 90° of elbow flexion. Study participants were asked to exert the maximum possible force on the dynamometer by squeezing the force exertion parts of the device with the dominant hand. Three individual measurements were taken at one-min intervals, and the mean of these three measurements was calculated and used for data evaluation. The results were expressed in kg. Grip strengths of <16 kg for females and <27 kg for males were considered in favour of diminished muscle strength [15]. Muscle performance was measured by grading gait speed along a 4-m track. The start and end of the track were marked distinctly so that the individual would be able to see them conveniently. After measuring the time required to complete the 4-m track using an electronic stopwatch, gait speed was calculated in m/s. The number of falls experienced by the individual in the last year was documented by asking the patients and their relatives.

The sleep time of the patients was categorised into two groups: <6 h and ≥6 h per night. The cut-off time of 6 h for the categorisation was determined after reviewing the results of similar previously published studies [17,18]. Sleep quality was classified as ‘good’ and ‘poor’ by asking the patient subjectively.

The patients’ frailty was assessed with the Canadian Study of Health and Aging Clinical Frailty Scale, in which high scores indicate frailty [19]. There are nine categories: 1: Very fit —robust, active, energetic, well-motivated, and fit; these people commonly exercise regularly and are in the most fit group for their age. 2: Fit—without active disease, but less fit than people in category 1. 3: Well, with the treated comorbid disease—disease symptoms are well controlled compared with those in category 4. 4: Apparently vulnerable—although not frankly dependent, these people commonly complain of being ‘slowed up’ or having disease symptoms. 5: Mildly frail—with limited dependence on others for instrumental activities of daily living. 6: Moderately frail—help is needed with both instrumental and noninstrumental activities of daily living. 7: Severely frail—completely dependent on others for activities of daily living, but not at high risk of dying within 6 months. 8: Very severely frail—completely dependent on others for activities of daily living and approaching the end of life. 9: Terminally ill—approaching the end of life with life expectancy <6 months. The ADL and IADL methods used in this scale were used as described above [19,20].

### 2.3. Laboratory assessment

Laboratory tests were performed in the morning with an open sample from the antecubital vein. Fasting blood glucose, creatinine, estimated glomerular filtration rate (eGFR), sodium, potassium, calcium, total protein, albumin, alanine aminotransferase (ALT), aspartate aminotransferase (AST), sedimentation rate, leukocyte count, haemoglobin, vitamin B12, folic acid, thyroid stimulating hormone (TSH), C-reactive protein (CRP) (mg/L), and 25-hydroxyvitamin D values were recorded. These values were added to the study because they were considered laboratory values that could be related to the general health, nutritional and inflammatory conditions of the patients. Biochemical parameters were studied using spectrophotometry. CRP was determined using the turbidimetric method, hormonal tests using the electrochemiluminescence immunoassay (ECLIA) method, and vitamin D levels using the high-performance liquid chromatography (HPLC) method.

### 2.4. Statistical analysis

The Statistical Package for the Social Sciences (SPSS) for Windows version 24.0 (IBM SPSS Inc., Chicago, IL) was used to perform the statistical analyses. The conformity of the variables to a normal distribution was examined using visual (histograms and probability graphs) and analytical (Kolmogorov-Smirnov/Shapiro-Wilk tests) methods. The results of the descriptive analyses were presented in mean and standard deviation for the normally distributed variables and in median and minimum-maximum for the nonnormally distributed variables. The frequency of the categorical variables was presented in percentages (%). The independent t-test was used to compare the means of two groups. The Mann-Whitney U test was used to compare two groups not conforming to a normal distribution. Multivariate logistic regression analysis was made with the variables of the duration of education, LB-IADL, MMSE, MNA-SF, sleep quality, sleep time, CFS, sedimentation rate, leukocyte count and CRP, which were significant in univariate linear regression analysis. Multivariate logistic regression analysis was performed to predict high depression scores status. As a result of the analysis, a significant regression model was obtained (*F* (8.148): 3.59, p: .001). It was found that 11% of the variance in the dependent variable (R^2^ adjusted: 0.11) was explained by the independent variables. Multivariate logistic regression analysis was performed using the enter method. Since there was no >0.8 value in Pearson correlation analysis and VIF values were not above 3, it was shown that there was no multicollinearity. The results were evaluated in a 95% confidence interval, and a p-value of < 0.05 was accepted as statistically significant.

## 3. Results

Of the 235 patients, the mean age was 73.6 ± 6.39 years, and 65.5% (n = 154) were women. The patients were divided into two groups according to the GDS-15 scores used in screening for depressive mood. Patients with a GDS-15 score of ≥5 were assigned to the ‘participants with higher depression rating scores group’ (34.9%, n = 82), and patients with a GDS-15 score of <5 were assigned to the ‘participants with lower depression rating scores group’ (65.1%, n = 148). The demographic and clinical data of the patients are summarised in Table 1. The number of medications was not significantly different between the two groups (p = .663).

The comparison of the patients according to the results of the comprehensive geriatric assessment, assessment of sleep characteristics, and frailty scores are summarised in Table 3.

In the participants with higher depression rating scores group, the LB-IADL, MMSE, and MNA-SF scores were low. Sleep quality was poor, and sleep time was short. The CFS scores indicating the vulnerability of the patients were high. In the patients in the participants with higher depression rating scores group, sedimentation rate, leukocyte count, and CRP values were found to be high. The laboratory values and their comparisons are summarised in Table 2.

As a first step, univariate linear regression analysis was performed with the variables found to be significant in Tables 1, 2, and 3. As shown in Table 4, duration of education, LB-IADL, MMSE, MNA-SF, sleep time, sleep quality, CFS, sedimentation rate, leukocyte count, and CRP were found to be significant predictive factors for higher depression scores. In the next step, multivariate logistic regression analysis was performed to predict higher depression scores status. According to the analysis results, the MMSE, MNA-SF, and CFS predicted higher depression scores significantly. Each unit of decrease in the MMSE score increased the risk of higher depression scores 0.8 times. Each unit of decrease in the MNA-SF score increased the risk of higher depression scores 0.6 times, and each unit of increase in the CFS increased the risk of higher depression scores 1.2 times.

## 4. Discussion

To our knowledge, this is the first study in Turkey to examine the relationship between depression scores, comprehensive geriatric assessment, and frailty in geriatric patients presenting to outpatient clinics with only somatic complaints.

The main findings of this study are that higher depression scores may be associated with instrumental activities of daily living, cognitive status, nutritional status, sleep duration, and quality and frailty in older people who presented to the geriatric outpatient clinic with somatic complaints who did not know that they had an underlying depression.

It was observed in this study that the rate of higher depression scores (34.9%) was quite high among outpatients; however, it was observed that these people were presented to geriatric outpatient clinics instead of seeing primary care physicians or psychiatrists. It is apparent that patients admitted to internal medicine outpatient clinics may have masked depressive mood underlying the aetiology of their unresolved somatic complaints. At this point, it is important to suspect that the clinical complaints of the patient might indicate depression, so depressive mood screening should be performed using relevant screening tools.

This study determined that the rate of higher depression scores was not different between genders. Consistent with that finding, some studies in the literature report no gender differences [21], but several other studies found higher rates of depression in women [2,4,22]. When the sociodemographic data of the patients in Table 1 were examined, no significant differences were found except for duration of education. When the groups of patients with and without higher depression scores were compared, a significant difference was found in educational status. Higher depression scores occurred at higher rates in patients who received less than 5 years of education. Additionally, the analysis revealed that a shorter duration of education in years was associated with depressive mood. Similar to these findings, some studies report that a shorter duration of education in years was a risk factor for developing depressive mood [23,24]. However, other studies reported just the opposite. Ashe et al. reported that the literacy rate in their study population was high, and there was no relationship between education level and severe depressive mood [22]. Another study did not find any relationship between education status and depression [25]. Further comprehensive research is needed on this subject.

The results of this study demonstrate that higher depression scores are associated with daily functioning (low LB-IADL scores), cognitive functions (low MMSE scores), and nutritional status (low MNA-SF scores) in older individuals. Previous studies have shown that these tests yielded similar scores within the scope of comprehensive geriatric assessment. In another study conducted in our country, female gender, sleep problems, and malnutrition were found to be independently associated with depression. [26–29]. These results reveal the extent of the physical effects of higher depression scores, which is often viewed as an entirely psychiatric condition in older individuals. It is noteworthy that the cognitive status determined by the MMSE and the nutritional status determined by the MNA-SF were negative predictors of higher depression scores, especially in the results obtained by regression analysis. These results substantiate the finding that depression should not be considered merely a psychiatric problem in older adults.

The laboratory results obtained in this study are notable because of the elevated levels of inflammation markers in the higher depression rating scores group. A review of existing studies examining the relationship between depression and inflammation revealed that some studies highlighted the relationship between depression and inflammatory markers. It has even been reported that antiinflammatory therapies can be used as a treatment option for depression [30–33]. As reported by these studies, depression may be an inflammation-related disease. Furthermore, considering the results of the present study, which demonstrated that the study population presented to clinics with physical issues, it may be suggested that these clinical manifestations are indicators of underlying inflammatory conditions. It would be appropriate to conduct further studies to clarify these observations by comparing depressed patients with individuals without physical complaints who have inflammation-related diseases.

In this study, the initial analysis found that sleep time was related to higher depression scores, but further analysis revealed that sleep time was not an associated factor of higher depression scores. Other studies investigating sleep and depression have shown that short sleep time and poor sleep quality lead to a depressed mood [34,35]. A meta-analysis reported that both short and long sleep times were related to depression [36]. The effect of depression on sleep may manifest as diminished sleep time and quality; however, it may also be a manifestation of anhedonia leading people to sleep to overcome dysphoria [37]. Diseases associated with sleep disorders have been found to be particularly related to obstructive sleep apnoea and depression.

In this study, CFS scores were higher in patients with higher depression scores. Further analyses also determined that frailty was a predictor for higher depression scores. Frailty is defined as a versatile loss of reserve in older people (energy, physical ability, cognition, and health) [19]. In this definition, among the criteria used to define frailty, daily basic and instrumental life activities are also included. Frailty also covers other criteria for being fit, including functionality. Some studies have shown that older people who are not physically and mentally fit may be prone to depression [38]. One study found that the presence of comorbidities that interact with depressive symptomatology increased the incidence of frailty [39]. A review of 84,351 older people emphasised that older people with depressive symptoms tend to be frail, and both conditions should be screened together. In addition, the treatment of possible depression could be a preventive measure for frailty if it is diagnosed in a timely manner [40]. Another study found that frailty and poor health were predictors of depressive symptoms [38]. As a result of these studies, it can be seen that each component of frailty includes a negative health condition that can make people depressed. This is a current issue in the agenda of geriatrics, and further research would be of interest.

The strengths of this study include the psychiatric distress assessment in patients presenting to internal medicine clinics and the comprehensive geriatric assessment. Another strength is the use of additional assessments of several potentially depression-related parameters other than psychiatric ones, including laboratory investigations.

There are some limitations to this study. First, it might have been more appropriate to evaluate sleep with more objective tools, such as the Epworth Sleepiness Scale, rather than asking patients questions. Secondly, the subtypes of depression (major depression, dysthymia, minor depression, psychotic depression, vascular depression, and depression comorbid with dementia) were not differentiated and no psychiatric assessment for depression, patients were grouped using screening scores. Since the geriatric scale of depression was used in the diagnosis, it would be more accurate to call this type subsyndromal. Finally, the study was conducted with patients admitted to a health institution based on their complaints, so it cannot be asserted that the results are completely representative of epidemiological findings of geriatric depression.

In conclusion, newly diagnosed higher depression scores in older people may be associated with not only mood but also the functionality. As with other geriatric syndromes, symptoms in higher depression scores may be atypical rather than typical. Even if older patients present with only somatic complaints, they should be screened for depression with appropriate tests.

## Figures and Tables

**Figure:**
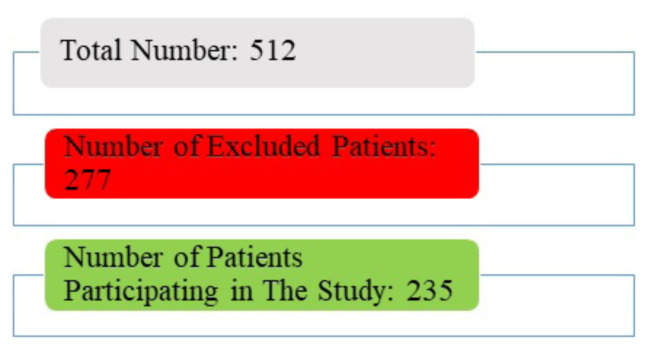
Total number of patients participating in the study (excluded patients who did not want to continue to perform a comprehensive geriatric assessment, had severe hearing problems, did not agree to perform full laboratory tests, and were unable to provide a clear response or suspected inconsistent response to sleep could not continue the study).

**Table 1:** Comparison of sociodemographic characteristics of patients.

	Total % (n)	Participants with lower depression rating scores % (n)	Participants with higher depression rating scores % (n)	p-value

	100% (n:235)	65.1% (n:148)	34.9% (n:82)	

Sex				
Female	65.8 (155)	41.0 (96)	24.8(58)	.116
Male	34.2 (80)	24.4 (57)	9.8 (23)	

Duration of education				
<5 years	48.5 (114)	27.0 (62)	21.3 (49)	**.004** ^**^
6–8 years	37.4 (88)	25.7 (59)	11.7 (27)	
> 8 years	14.0 (33)	11.7 (27)	2.6 (6)	

Smoking				
No	60.9 (143)	47.9 (95)	22.8 (46)	.357
Yes	26.8 (63)	18.3 (37)	11.9 (24)	

Marital Status				
Married	46.8 (110)	35.7 (70)	18.9 (37)	.841
Single	38.3 (90)	29.1 (57)	16.3 (32)	

Living status				
Alone	24.7 (58)	19.2 (38)	9.6 (19)	.597
With spouse	35.7 (84)	27.3 (54)	14.1 (28)	
With children	14.9 (35)	11.3 (23)	6.1 (12)	
With spouse and children	10.6 (25)	7.1 (14)	5.1 (10)	

Hypertension				
No	27.7 (65)	17.4 (40)	10.4 (24)	.415
Yes	72.3 (170)	47.2 (108)	25.2 (58)	

Diabetes mellitus				
No	58.3 (137)	36.1 (83)	21.7 (50)	.472
Yes	41.7 (98)	28.3 (65)	13.9 (32)	

COPD				
No	84.7 (199)	56.5 (130)	28.3 (65)	.083
Yes	15.3 (36)	7.8 (18)	7.4 (17)	

Heart failure				
No	90.2 (212)	59.6 (137)	30.4 (70)	.081
Yes	9.8 (23)	4.8 (11)	5.2 (12)	

Dementia				
No	92.8 (218)	60.9 (140)	31.7 (73)	.122
Yes	7.2 (17)	3.5% (n = 8)	3.9 (9)	

Cancer				
No	91.9 (216)	59.6 (137)	32.2 (74)	.540
Yes	8.1 (19)	4.8 (11)	3.5 (8)	

Cerebrovascular event				
No	93.2 (219)	59.6 (137)	33.5 (77)	.703
Yes	6.8 (16)	4.8 (11)	2.2 (5)	

Hypothyroidism				
No	84.7 (199)	53.5 (123)	30.9 (71)	.487
Yes	15.3 (36)	10.9 (25)	4.8 (11)	

Chronic kidney disease				
No	93.6 (220)	60.4 (139)	33.5 (77)	.996
Yes	6.4 (15)	3.9 (9)	2.2 (5)	

* Percentages in cells show percentages in the total. Missing values are included in the calculation of the percentages.

(Abbreviations: COPD: Chronic Obstructive Pulmonary Disease)

**Table 2:** Comparison of the laboratory values of the study patients.

	Participants with lower depression rating scores Median (min–max)	Participants with higher depression rating scores Median (min–max)	p-value
Fasting blood glucose (mg/dL)	116.0 (74.0–462.0)	110.0 (90.0–355.0)	.304
Creatinine (mg/dL)	0.82 (0.47–1.56)	0.93 (0.55–2.76)	.636
Sodium (mmol/L)	139.0 (127.0–143.0)	139.0 (132.0–143.0)	.616
Potassium (mmol/L)	4.50 ± 0.39	4.55 ± 0.45	.820
Calcium (mg/dL)	9.90 ± 0.50	9.76 ± 0.64	.067
Albumin (g/L)	4.15 ± 0.28	4.15 ± 0.21	.087
Alanine Aminotransferase (ALT) (U/L)	20.58 ± 15.56	15.69 ± 6.64	.149
Aspartate Aminotransferase (AST) (U/L)	21.0 (8.0–88.0)	18.0 (3.0–71.0)	.129
Sedimentation (mm/h)	20.32 ± 14.59	24.93 ± 15.00	**.036** *
Leukocyte (WBC) (x10^9/L)	7.54 ± 1.92	8.01 ± 2.16	**.048** *
Hemoglobin (Hb) (g/dL)	13.2 (8.6–16.4)	12.7 (10.2–15.6)	.053
Platelet Count	279.19 ± 80.65	298.54 ± 62.68	.112
HbA1c	7.47 ± 1.88	7.31 ± 2.24	.725
Vitamin B12 (pg/mL)	420.48 ± 332.10	435.48 ± 378.44	.528
TSH (μIU/mL)	1.89 ± 1.19	3.98 ± 12.39	.070
CRP (mg/L)	6.47 ± 10.76	9.00 ± 13.32	**.043** *
25-Hydroxy vitamin D (μg/L)	18.8 (5.20–46.52)	16.2 (5.40–45.50)	.667

* The results of the descriptive analyses were presented in mean and standard deviation for the normally distributed variables, and in median and maximum for the nonnormally distributed variables (Abbreviations: TSH: thyroid-stimulating hormone; CRP: C-reactive protein).

**Table 3:** Comparison of the study patients by the comprehensive geriatric assessment results.

	Participants with lower depression rating scores median (min–max)	Participants with higher depression rating scores median (min–max)	p-value

Katz ADL	6 (2–6)	6 (1–6)	,420

LB-IADL	8 (1–8)	6 (1–8)	**.010** *

MMSE	26 (13–30)	23 (10–30)	**<.001** *

MNA-SF	13 (10–14)	11 (10–14)	**.003** *

Gait speed (m/s)	7 (5–30)	7 (5–19)	.163

Number of falls	0 (0–3)	0 (0–3)	.094

Sleep quality			
Poor	32.2% (n = 74)	59.6% (n = 137)	**.006** *
Good	4.8% (n = 11)	3.5% (n = 8)	

Sleep time			
<6 h	32% (n = 18.9)	17.2% (n = 29)	**.042** *
≥6 h	46.2% (n = 78)	17.8% (n = 30)	

Clinical Frailty Score	Median (min–max)		**.035** *

	4 (2–7)	5 (2–7)	

* The results of the descriptive analyses were presented in mean and standard deviation for the normally distributed variables, and in median and maximum-minimum for the nonnormally distributed variables.

(Abbreviations: Katz ADL: Katz index of activities of daily living; LB-IADL: The Lawton-Brody instrumental activities of daily living scale; MMSE: The Mini-Mental state exam; MNA-SF: Mini nutritional assessment-short-form).

**Table 4:** Univariate linear and multivariate logistic regression analysis of associated factors of depression.

	Univariate analysis			Multivariate analysis		
	Odd Ratio	p-value	95% C.I.	Odd Ratio	p-value	95% C.I.
Duration of education (<5 years)	3.551	**.010**	1.361–9.295	2.197	.236	.598–8.079
LB-IADL	.873	**<.001**	.802–.951	.887	.137	.757–1.039
MMSE	.889	**<.001**	.831–.948	.892	**.048**	.798–.999
MNA-SF	.696	**.012**	.525–.923	.661	**.045**	.441–.991
Sleep time (<6 h )	2.356	**<.001**	1.223–4.539	1.747	.203	.740–4.125
Sleep quality (Poor)	2.500	**.006**	1.295–4.826	1.483	.403	.589–3.745
Clinical frailty score	1.321	**.016**	1.052–1.659	1.245	**.047**	1.046–1.578
Sedimentation (mm/h)	1.017	**.036**	1.001–1.033	1.005	.640	.984–1.027
Leukocyte (WBC) (x10^9/L)	1.116	.099	.980–1.272	-	-	-
CRP (mg/L)	.997	.724	.983–1.012	-	-	-

(Abbreviations: LB-IADL: The Lawton-Brody instrumental activities of daily living scale; MMSE: The Mini-Mental state exam; MNA-SF: Mini nutritional assessment-short-form; CRP: C-reactive protein; CI: Confidence Interval).
